# Supplementary material for: Challenges in detection and treatment of multidrug resistant tuberculosis patients in Vietnam
Source: BMC Public Health. 2015 Sep 29;15:980. doi: 10.1186/s12889-015-2338-5 (PMC4587724; doi:10.1186/s12889-015-2338-5)
Supplement: Additional file 1: — Definitions and Interview guide (DOCX 28 kb) [file 12889_2015_2338_MOESM1_ESM.docx]

Challenges in detection and treatment of multidrug resistant tuberculosis patients in Vietnam

Hoang Thi Thanh Thuy[1*], *corresponding author; Email: hoangthanht@gmail.com

Nguyen Viet Nhung[1], Email: vietnhung@yahoo.com

Dinh Ngoc Sy[2], Email: syminhquan@gmail.com

Nguyen Binh Hoa[1,3], Email:nguyenbinhhoatb@yahoo.com

Frank Cobelens[4], Email: f.cobelens@amc-cpcd.org

Guy Thwaites[5,6] Email: gthwaites@oucru.org

Nguyen Thien Huong[7]: nthuong139@gmail.com

Nguyen Thu Anh[8], Email: thuanh.nguyen@sydney.edu.au

Pamela Wright[9], Email:pamela.wright@mcnv.nl

Heiman F.L. Wertheim[6,10,11] Email: heiman.wertheim@gmail.com

1. National Tuberculosis Control Program of Vietnam- National Lung Hospital (VNTP-NLH)
2. Vietnam Association for Tuberculosis and Lung Disease, Hanoi, Vietnam
3. Centre for Operational Research, International Union Against Tuberculosis and Lung Disease, Paris, France
4. Department of Global Health and Amsterdam Institute for Global Health and Development, Academic Medical Center, Amsterdam, Netherlands
5. Wellcome Trust Major Overseas Program, Oxford University Clinical Research Unit, Ho Chi Minh City, Vietnam
6. Nuffield Department of Clinical Medicine, Centre for Tropical Medicine, University of Oxford, United Kingdom.
7. KNCV Tuberculosis Foundation, Vietnam Country Office, Hanoi, Vietnam
8. Woolcock Institute Of Medical Research, Australia
9. Medisch Comite Nederland-Vietnam, Netherlands
10. Wellcome Trust Major Overseas Program, Oxford University Clinical Research Unit, Hanoi, Vietnam (OUCRU)
11. Department of Medical Microbiology, Radboudumc, Nijmegen, Netherlands

Abstract

Background: Vietnam is ranked 14th among 27 countries with high burden of multidrug-resistant tuberculosis (MDR-TB). In 2009, the Vietnamese government issued a policy on MDR-TB called Programmatic Management of Drug-resistant Tuberculosis (PMDT) to enhance and scale up diagnosis and treatment services for MDR-TB. Here we assess the PMDT performance in 2013 to determine the challenges to the successful identification and enrollment for treatment of MDR-TB in Vietnam.

Methods: In 35 provinces implementing PMDT, we quantified the number of MDR-TB presumptive patients tested for MDR-TB by Xpert MTB/RIF and the number of MDR-TB patients started on second-line treatment. In addition, existing reports and documents related to MDR-TB policies and guidelines in Vietnam were reviewed, supplemented with focus group discussions and in-depth interviews with MDR-TB key staff members.

Results: 5,668 (31.2%) of estimated 18,165 MDR-TB presumptive cases were tested by Xpert MTB/RIF and second-line treatment was provided to 948 out of 5,100 (18.7%) of MDR-TB patients. Those tested for MDR-TB were 340/3,224 (10.5%) of TB-HIV co-infected patients and 290/2,214 (13.1%) of patients who remained sputum smear-positive after 2 and 3 months of category I TB regimen. Qualitative findings revealed the following challenges to detection and enrollment of MDR-TB in Vietnam: insufficient TB screening capacity at district hospitals where TB units were not available and poor communication and implementation of policy changes. Instructions for policy changes were not always received, and training was inconsistent between training courses. The private sector did not adequately report MDR-TB cases to the NTP.

Conclusions: The proportion of MDR-TB patients diagnosed and enrolled for second-line treatment is less than 20% of the estimated total. The low enrollment is largely due to the fact that many patients at risk are missed for MDR-TB screening. In order to detect more MDR-TB cases, Vietnam should intensify case finding of MDR-TB by a comprehensive strategy to screen for MDR-TB among new cases rather than targeting previously treated cases, in particular those with HIV co-infection and contacts of MDR-TB patients, and should engage the private sector in PMDT.

Key words: Multi drug resistant tuberculosis, detection, treatment, Vietnam

Background

Multidrug-resistant tuberculosis (MDR-TB) is a global health concern as treatment is prolonged, costly, and less effective compared to that of drug-susceptible TB. Globally, there are about 450,000 MDR-TB patients reported with an estimated 170,000 MDR-TB-related deaths annually. Regarding the TB and MDR-TB epidemiology in Vietnam, the country is ranked 12nd among 22 high burden countries with TB, and 14th among 27 countries with a high burden of MDR-TB. The incidence of TB in Vietnam is estimated at 144 per 100,000 population per year and the estimated prevalence is 209 cases per 100,000 population[1]. There are estimated to be about 5,100 MDR-TB cases among notified TB cases per year. The proportion of TB cases with MDR-TB among new and retreatment cases is estimated to be 4% and 23%, respectively, with around 6% among TB cases co-infected with HIV[1]. A preliminary report from a TB drug resistance survey conducted in Vietnam from 2011-2012 showed that the estimated proportion of XDR-TB among MDR-TB was 5.6%. Of the MDR-TB patients, 16.7% showed resistance to fluoroquinolones (ofloxacin), 1.1% to amikacin, 5.6% to kanamycin, and 5.6% to capreomycin (unpublished data).

In 2009, the Vietnamese government issued a policy on MDR-TB following WHO recommendations to enhance and scale up diagnosis and treatment services for MDR-TB [2]. It is based on the five essential control components that constitute the already implemented DOTS strategy for drug-susceptible TB. These components include: sustained political commitment, rational case finding, short-course treatment, an uninterrupted drugs supply, and standardized recording and reporting[3]. Since a pilot project in Ho Chi Minh City in 2009, diagnosis and treatment services for MDR-TB have become available in 35 of all 63 provinces by 2013.The programmatic management of drug-resistant tuberculosis (PMDT) is more complex than for susceptible TB as it requires greater human, financial and technical resources [3]. Between 2009 and 2011 the Vietnam PMDT program had a success rate, defined as MDR-TB patients who completed treatment and were cured, of approximately75%. This rate is high compared to many other countries reporting rates of 44-58%[4][5]. Despite that the treatment outcome may depend on drug resistance patterns, this high success rate does reflect good compliance with the treatment regimen in Vietnam[6]. The current standard second-line regimen is kanamycin, capreomycin, levofloxacin, cycloserine, protionamide, and para-aminosalicyclic acid (PAS).

However, the number of MDR-TB patients detected and enrolled to second-line treatment is low compared to the expected number among notified TB cases, based on national drug susceptibility data [7]. This means that only a few of the MDR-TB patients get diagnosis and the recommended treatment. This can partly be explained by the fact that Vietnam did not have nation-wide PMDT coverage by 2013, and drug susceptibility testing was not yet done for all notified TB cases. However, PMDT was fully implemented in major cities by 2010, where just 30-50% of the total estimated MDR-TB patients were enrolled for second-line treatment. This finding lead us to assess the proportion of MDR-TB patients enrolled out of the estimated number of MDR-TB in all PMDT areas of Vietnam in 2013 to determine the challenges to an efficient PMDT implementation and to provide recommendation to improve the PMDT enrollment.

PMDT includes five steps from diagnosis to treatment for MDR-TB patients:(1) identification of presumptive MDR-TB cases (individuals considered at high risk for MDR- TB) according to case definitions, (2) referring presumptive cases for diagnosis, (3) drug resistance testing, (4) obtaining informed consent from patients for treatment, and (5) enrollment of diagnosed MDR-TB patients to treatment. In this assessment, we used the data reported by the national TB control program to focus on three of these steps: identification of presumptive MDR-TB cases, drug resistance testing, and enrollment of diagnosed MDR-TB patients for treatment. Qualitative investigation was used for assessment of steps (2) and (4) and additionally to support the findings and their interpretation for the other steps.

Methods

By 2013, 35 provinces had implemented the PMDT and participated in the MDR-TB enrollment assessment. Thirty-one provinces were selected by the NTP to implement PMDT based on their prior MDR-TB case-load and case management capacity. The remaining four provinces with a low MDR-TB case-load were selected as they had a high HIV prevalence. All 35 provinces were provided access to diagnostic equipment for intensified case finding using the Xpert MTB/RIF (Xpert) assay, a within-cartridge real-time PCR assay that detects *M. tuberculosis* as well as mutations in the *rpoB* gene conferring resistance to rifampicin in clinical specimens[8]. The selected provinces are required to provide PMDT treatment services in case MDR-TB patients are

diagnosed. For the assessment, we reviewed all existing reports and documents related to MDR-TB policies and guidance since 2009 through December 2013.

A presumptive MDR-TB case requiring testing was defined as belonging to at least one of the risk groups as defined in the NTP guidelines (see supplementary materials). In order to calculate the proportion of those screened for MDR-TB, we divided the number of tested presumptive MDR-TB cases by the number of presumptive MDR-TB cases estimated using annual data reported by each province to the NTP (step1). All PMDT provinces provide quarterly reports to the NTP of the number of Xpert MTB/RIF tests done and the number of MDR-TB cases detected. We used the 2013 Xpert MTB/RIF data for assessing the number of presumptive cases screened per risk category (step 3).

For national estimation of MDR-TB, we used the number of all notified TB cases per province and applied the result of the most recent national DRS in Vietnam (year 2011-2012) which showed prevalence of MDR-TB among new and previously treated cases of 4.0% and 23.0%, respectively[1]. This allowed us to estimate the expected number of incident MDR-TB cases in all provinces irrespective of detection by the NTP. We used the standard errors from the MDR-TB prevalence as estimated in the DRS to calculate confidence intervals for the proportion of MDR-TB patients enrolled for second-line treatment in this study. The proportion with 95% confidence intervals (95% CI) was estimated for the number of MDR-TB patients enrolled for second-line treatment among (i) the estimated number of MDR-TB cases in the whole country and (ii) the estimated number of MDR-TB cases in the 35 PMDT provinces (step5).

This review was followed by focus group discussions (FGD) in face-to-face workshops, which encompassed five separate one-day sessions with approximately 30 key provincial TB staff members (for all PMDT steps including steps 2 and 4). FGD were used to discuss the PMDT program, challenges to implementation and potential solutions. Five groups were invited from eight different geographic areas (see supplementary materials). TB staff included provincial program managers, program officers with expected knowledge of the local overall situation and staff responsible for implementation of PMDT in their province. Guidance was provided for FGD facilitators who had previous FGD discussion experience and knowledge of the NTP. Discussions

were continued until saturation was reached before facilitators moved to a new topic. FGD summaries were checked and agreed upon by all participants to be finalized for content analysis later on. The sessions were not audio recorded and transcribed due to lack of funds.

Furthermore, in-depth interviews to supplement the focus group discussions was conducted by the same group of interviewers with eighty TB health staff at different levels of the NTP, including 8 central staff from PMDT team, 56 provincial staff from variety of departments, and 16 district and commune level health staff (for all steps). The interview guide was developed and piloted before the interviews and included questions regarding: structure of the TB health unit at specific levels in the health system, training provided to staff, their knowledge and awareness about MDR-TB risk groups, their role and job description, and challenges encountered in PMDT implementation. The in-depth interview also allowed the interviewee to reflect on findings from the FGD. These interviews were conducted during monitoring visits in 2014 in eight randomly selected provinces among the 35 PMDT provinces from three zones: four provinces were randomly selected from the southern zone as there exists the highest disease burden, two from the north and two from the centre (see supplementary information). The interviews were conducted privately. During the interview and FGDs, detailed notes were taken and used subsequently to identify key themes. In the analysis, similarities and differences between the various interviews and FGDs were looked for and summarized to identify key findings. The interviews were not recorded.

This study was approved by the research and ethics committee of the National Lung Hospital in Hanoi. Informed consent was obtained from participants in workshops and in-depth interviews.

Results

The estimated number of MDR-TB cases in Viet Nam

Based on the notification report of the NTP in 2013 and the results of the recent national DRS, we estimated that in 2013 there were 5,065 (95% CI: 3,355-6,700) MDR-TB patients among 102,196 notified TB cases, resulting in a proportion of 5.0% MDR-TB among notified cases for the year 2013 (95% CI: 3.3%-6.6%). These cases were mainly concentrated in the South–East, Mekong Delta and Red River Delta regions. The PMDT program already covered provinces with an expected high number of MDR-TB cases (Figure 1 and Table 1). We estimated that the majority

3,982/5,065 (78.6%) of the national MDR-TB case-load originates in the provinces participating in the PMDT (table 1).

Identification of MDR-TB presumptive cases to be tested (step 1)

In 2013, there were an estimated 18,165 cases identified as presumptive MDR-TB nationally, with the majority (n=14,998) in the 35 PMDT provinces (82.6 %). There was no data available on the number of MDR-TB presumptive cases identified by the health staff of PMDT areas who were referred for screening.

Testing practices per risk category by Xpert MTB/RIF and detection of MDR-TB (step 3)

A total of 32 Xpert MTB/RIF instruments were distributed among the 35 PMDT provinces. The estimated number of MDR-TB cases to be detected per machine per year for these instruments varied by region, ranging from 9 to 338 MDR-TB cases (see supplementary material). The instrument's capacity is expected to be sufficient as one instrument with four modules is able to test approximately 3,000 MDR-TB presumptive cases to detect around 500 rifampicin resistant TB cases per year (see supplementary material).

The proportion of MDR-TB presumptive cases tested by Xpert MTB/RIF was 31.2% (5,668/18,165) for the whole country and 37.8% (5,668/14,998) in the 35 PMDT provinces (Table 2). In PMDT provinces, the highest proportion of MDR-TB presumptive cases tested was in the North-West (43/46; 93.9%) and the Southern Central Coast (668/897; 74.5%) regions. The lowest proportions were in the Central Highlands (0/69; 0%) and the Mekong Delta (516/3935; 13.1%; Table 2) regions. In the North-West region, all provinces together only had 43/214 (20.1%) of presumptive cases tested while that percentage was 43/46 (93.9%) in the PMDT provinces. Among 5,668 MDR-TB presumptive cases tested, 997 cases were detected as rifampicin resistant (17.6%).

The proportions of HIV infected TB cases and new TB non-converters tested by Xpert MTB/RIF were 10.5% (340/3,224) and 13.1% (290/2,214), respectively (Table 3). Retreatment patients who showed no sputum conversion after 3 months of the category II regimen were tested frequently: 85.7% (413/482). We estimated that 249/402 (62.0%) of symptomatic household contacts of MDR-TB patients were tested. Among 249 MDR-TB symptomatic household contacts screened within 1 year after MDR-TB patients diagnosed, 77 TB patients were detected (30.9%) including 9

TB patients resistant to rifampicin (3.6%). We could not calculate the testing rate among failure cases since no data were available for the number of people tested. However, this rate was estimated to be at least 68.5% because one failure case could receive up to two tests.

Enrollment to second-line treatment (step 5)

Of 997 rifampicin-resistant cases detected, 948 (95.1%) were enrolled for MDR-TB treatment, accounting for just 18.7% (95% CI: 14.1%-28.3%) of the estimated 5,065 MDR-TB cases in the whole of Vietnam (Figure 2). The region with the highest proportion of MDR-TB patient enrolled for second-line treatment was the South-East region with 495/1,546 (32.0%; 95% CI: 24.3%-47.8%), followed by the Red River Delta with 234/859 (27.3%; 95% CI: 20.5% -41.4%). The lowest enrollments were in the Mekong Delta region: 81/1,288 (6.3%; 95% CI: 4.8%-9.5%). No enrollments were reported in PMDT for North-East, North-West and Central Highlands (Table 1, Figure 3). Also in the 35 PMDT provinces, the enrollment proportion of MDR-TB cases was low: 948/3,982 (23.8%; 95% CI:18.0%-35.9%). The highest proportion was seen in the PMDT provinces in the Red River Delta with 234/627(37.3%; 95% CI: 28.2%-56.5%), and South-East region: 495/1,408 (35.2%; 95% CI: 26.7%-52.4%)

Qualitative investigations (all steps)

This section reveals the challenges to a successful diagnosis and enrollment for treatment of MDR-TB (table 4). It was noted that TB units (i.e. small departments operating under the NTP that identify MDR-TB presumptive, provide TB diagnostic and first-line treatment services by specifically trained staff) are located mainly in district health centers. Although there is a policy change ongoing to have TB units also in (district) hospitals this had not yet been implemented in all districts. As a result, several district hospitals, where many MDR-TB presumptive cases present did not have TB diagnostic facilities and hospital staff had not been trained and instructed to screen patients for TB and MDR-TB.

Provincial and district staff reported that they were overall not well informed about PMDT procedures. The first guideline for PMDT was issued in 2009 [2] and has not been updated for any of the multiple changes since then. The changes in policies over time had been updated through

separate instruction letters from the central level and though yearly PMDT training courses without documenting these in a clear set of guidelines. A total of 17 instruction letters to inform about changes have been sent out from central level to provinces between 2009 to late 2013. However, regularly these update letters were not received by the provincial PMDT officer or the information was not disseminated to the relevant staff at lower levels. A further challenge was regular health care staff turnover or change in health care staff's responsibilities over time. As a consequence, it is challenging to ensure training and skills development for all the staff involved in TB and MDR-TB management.

Standard operating procedures (SOPs) for PMDT were developed and released in 2012, three years since the MDR-TB guideline was released. The SOPs lacked clear instructions for steps that need to be taken[9]. No standard PMDT training modules are available as the training and presentations were prepared and updated by different lecturers, based on their interpretation of the available guidelines and SOPs. Inconsistencies across training courses were mentioned by trained health staff, which generated uncertainty. In addition, the majority of district level staff was not trained due to lack of funding. In 2013, the majority of district staff with an assigned responsibility to identify presumptive MDR-TB had not been trained to identify risk groups. Supervision visits conducted between April and July 2014 revealed that, although all districts under PMDT had been trained, their focus was on retreatment cases, while non-converters among new cases and HIV-positive TB patients were generally neglected.

Many TB units were unable to send sputum for testing by Xpert MTB/RIF as no appropriate financial compensation mechanisms were in place for consumable procurement and sputum transportation fees. Partly this was due to a delay in an award from the Global Fund. In addition it was not straightforward to send specimens by mail as no agreements with the postal services were in place, and sputum referral using health staff or public transportation were used instead. This may explain why in some regions with Xpert MTB/RIF capacity, like the Mekong delta region, the proportion tested and enrolled was limited. Finally, there had been stock outs of Xpert MTB/RIF test kits due to delays in cartridge forecast reporting. Although challenges were mentioned for sending sputum, there was always a confirmation and communication between referring sites and laboratory after samples had been sent. A small number of referred samples were rejected by the

laboratory for being considered low-quality or low-volume, and requested to re-collect and re-send.

For MDR-TB patients that were diagnosed, health staff mentioned that informed consent was obtained for all of these patients before starting treatment. There were issues regarding enrolling MDR-TB patients for treatment due to lack of hospitalization capacity. It was reported that poor links existed between the public and private sector, as patients cannot be referred from the private to the public sector to continue their treatment. Furthermore, there are no management systems in place to screen and treat MDR-TB patients in prison (see Table 4 for more details).

Discussion

We estimated that only one third of MDR-TB presumptive cases are screened by Xpert MTB/RIF in Vietnam. However, it is encouraging that 95% of the patients who were tested positive for MDR-TB were enrolled for second-line treatment. The low enrollment rate is mainly attributable to low number of presumptive MDR-TB cases identified and subsequently tested, and not due to poor enrollment of patients after diagnosis. There were some inconsistencies between the percentage of MDR-TB presumptive cases tested in 35 PMDT provinces (table 2) and the enrollment rate of MDR-TB (Table 1). While there were relative high testing proportion in the North-East, North-West, Northern Central and Southern Central Coast regions, the enrollment proportion for treatment among number estimated were low, varying from 0% to 27.3%. In other regions, the variations in enrollment rates were in line with the variations in percentage of MDR-TB presumptive cases tested. The reasons behind these inconsistencies were limited capacity for hospitalization and other issues as revealed by our qualitative assessment.

This study showed that access to Xpert MTB/RIF testing was overall sufficient for Vietnam. However, regions with a higher number of operational Xpert MTB/RIF instruments had a higher enrollment proportion compared to regions with fewer instruments, except for the Mekong River Delta (figure 3). While the burden of MDR-TB in Mekong Delta region was high, with Xpert MTB/RIF instruments widely available, the enrollment proportion in this region was very low: 7.5% (Table 1, Figure 3). The reason behind the low enrollment proportion in Mekong Delta region is a weak system for transferring specimens for Xpert MTB/RIF testing as revealed by our

qualitative study. In addition, procurement of test kits needs improvement to avoid stock outs, and redistribution of Xpert MTB/RIF instruments from the Red River Delta with 9 instruments and a low workload to the Northern Central region with just one instrument and a high workload may improve access to testing for risk categories. Testing coverage was nil in the Central Highlands region. Although this area has a relatively low number of estimated MDR-TB cases, facilitating a good specimen referral system for testing could increase the coverage. Main challenges for effective MDR-TB diagnosis and treatment and potential solutions proposed by our study team for under enrollment are listed in Table 4.

New TB patients whose sputum had not converted after 2 or 3 months of treatment and TB patients with HIV, both considered risk groups for MDR-TB, were largely neglected for screening and had the lowest proportions of MDR-TB screening done while they account for the majority of presumptive MDR-TB cases[2][3]. Since the proportion of detected rifampicin-resistant patients among these groups in 2013 was 11.3% [10], it is likely that a considerable number of MDR-TB remain undiagnosed due to inadequate screening among these groups. Importantly, undetected MDR-TB/HIV patients who have high mortality rates [11][12] would lose the opportunity for adequate treatment. Moreover, TB-HIV patients in 2013 are underreported due to limited collaboration between the TB and HIV control programs [13].

The qualitative assessment revealed that many patients refuse treatment in units far from home. Other patients reported problems getting health insurance reimbursement in case they were referred to another province. Lack of collaboration between treatment centers and adjacent provinces for referring patients for treatment also contributed to drop outs. Another contributing factor to treatment delays and poor enrollment was the irregular delivery and stock outs of MDR-TB treatment drugs due to procurement delays. Occasionally, patients insisted to be enrolled, but they had to wait for a long time to start treatment.

This study also revealed poor links between the public and private sector. Due to absence of a referral mechanism between the two systems, MDR-TB presumptive were not referred from private sector to the NTP for diagnosis, and patients who withdrew from MDR-TB treatment in the private sector for financial or other reasons were unable to enroll to continue treatment in the

public PMDT. Currently, only limited data for MDR-TB patients in the private sector are available, where default rates are high, up to 75%, implying poor MDR-TB management in the private sector that may lead to drug resistance amplification[14]. Also MDR-TB patients in prisons pose a challenge as second-line treatment is not available, and no procedures for management of MDR-TB patients after discharge from prison are in place.

The current PMDT policy is that each district should have a TB unit. The Ministry of Health has as a policy to locate TB units in general hospitals, or in district health centers if a general hospital is not present [15]. This policy is aimed at strengthening the utilization of diagnosis and treatment services for TB units in the district hospital, where most patients seek health care. However, our study found that not all district hospitals have a TB unit with TB screening capacity. Health staff lack proper training and MDR-TB screening skills and do not have access to updated guidelines/SOPs to support them in identifying patients. This results in confusion and limited confidence among staff to implement the PMDT properly. Inconsistencies across training courses also contributed to confusion around the PMDT policies. Clear guidelines, instructions and training for health staff is needed to improve this.

Our study has several limitations. To estimate the MDR-TB enrollment proportion we used two different data sets: the 2013 NTP report and the DRS conducted in 2011-2012. For the number of MDR-TB cases detected, we only used laboratory reports from Xpert MTB/RIF testing and we did not include reports from other tests such as line-probe assays or phenotypic drug susceptibility testing. As the number of tests done by these other methods is small, less than 5% with considerable overlap with Xpert MTB/RIF testing, we decided to exclude these.

Furthermore, as we do not know the prevalence of rifampicin resistance for each MDR-TB risk group we could not assess the positive predictive value (PPV) of Xpert MTB/RIF separately among each category of presumptive MDR-TB patients. However, for most categories, the estimated prevalence of rifampicin resistance is relative high (around one-fourth, given the 23% prevalence among previously treated patients in the DRS). In addition, recent studies from South Africa[16] and Brazil [17] suggest a PPV on the order of 90% even with relatively low prevalence of rifampicin resistance. Therefore we do not expect that the PPV in our study will have been much below 90%, and thus did not affect the results to considerable extent.

A final limitation is that we did not involve the private sector in our study. However, our qualitative assessment suggests poor management of MDR-TB in the private sector and a lack of a good referral system between the private and public sector. This situation causes patients to be under screened, poorly treated, and underreported to the NTP. Better links need to be established between the private and public sector.

Conclusion

The proportion of MDR-TB patients enrolled for second-line treatment among the total estimated number of MDR-TB cases in Vietnam in 2013 was 18.7% (948/5,065). The low enrollment was considered due to under-screening of MDR-TB presumptive cases, especially for HIV infected and new TB non-converters. Multiple reasons exist for under-screening, including: poor communication and implementation of policy changes, lack of involvement of general district hospitals, and limited resources. However, Vietnam has achieved a high proportion of enrollment to second-line treatment among of detected MDR-TB cases and a high treatment success rate, which can be considered an opportunity to mobilize resources and expand the program to detect and treat more MDR-TB cases. Vietnam should expand the intensified case finding of MDR TB by a comprehensive strategy with more focus on new detected cases, in particular those with HIV co-infection and contacts of MDR-TB patients. The capacity to treat new MDR-TB cases will need to be increased in case the screening is improved. The private sector needs to be engaged in the PMDT.

Competing interest

The authors declare that they have no competing interests.

Authors' contributions

NVN, HTTT, HW, NTA conceptualized and designed the study. HTTT was responsible for data collection, analysis and interpretation of data. HTTT wrote the first draft of the paper with critical advice provided by NVN, HW, NTA, PW, NTH, NBH and FC. All authors reviewed and approved the manuscript as submitted

Acknowledgments

This work was approved by the NTP to be conducted and financially supported by the Wellcome Trust Major Overseas Program, Vietnam. The sponsor had no role in the study design, data collection, analysis or decision to submit for publication. Thanks to Mr. Tran Nhu Phong, Woolcock Institute of Medical Research, for his help to structure the maps for manuscript.

REFERENCES

[1] World Health Organization: *Global tuberculosis report 2014*. Geneva, Switzerland: WHO; 2014. WHO/HTM/TB/2014.08.

[2] National Tuberculosis Control Program of Vietnam : *National Guidelines for Programmatic Management of Drug Resistance Tuberculosis.* Hanoi: Ministry of Health; 2009: 1-142.

[3] World Health Organization:*Guidelines for the programmatic management of drug-resistant tuberculosis*. Geneva, Switzerland: WHO; 2008.WHO/HTM/TB/2008.402

[4] World Health Organization: *Global tuberculosis report 2013*. Geneva, Switzerland: WHO; 2013. WHO/HTM/TB/2013.11

[5] World Health Organization: *Global tuberculosis report 2012*. Geneva, Switzerland: WHO; 2012. WHO/HTM/TB/2012.6

[6] C. Nagaraja, B. L. Shashibhushan, M. Asif, M. Ph, and C. Sagar:**Pattern of Drug-resistance and Treatment Outcome in Multidrug-resistant Pulmonary Tuberculosis**. *Indian J Chest Dis Allied Sci*. 2012 Jan-Mar; 54(1):23-6.

[7] R. M. Falzon D1, Jaramillo E, Wares F, Zignol M, Floyd K: **Universal access to care for multidrug-resistant tuberculosis**: **an analysis of surveillance data**. *Lancet Infect Dis,* 2013, 13(8):690-7. doi: 10.1016/S1473-3099(13)70130-0. Epub 2013 Jun 4

[8] World Health Organization: *Rapid Implementation of the Xpert MTB / RIF diagnostic test, Technical and Operational "How-to", Practical consideration.* Geneva, Switzerland: WHO; 2011. WHO/HTM/TB/2011.2

[9] National Tuberculosis Control Program of Vietnam : *Standard Operational Procedure for Programatic Management of Drug resistant Tuberculosis in Vietnam.* Hanoi: Ministry of Health; 2012: 1-86

[10] National Tuberculosis Control Program of Vietnam : *Laboratory performance report from National TB control programme of Vietnam.* Hanoi: National Reference Laboratory; 2013. Ref Type: Report.

[11] Finlay AF: **Treatment outcomes of patients with multidrug resistant tuberculosis in South Africa using a standardized regimen, 1999–2000**. I*nfectious Disease Society of America* (Poster session 58, N_ 2101). Boston, MA, September 2004.3.

[12] Park MM, Davis A L, Schluger N W, Cohen H, Rom WN: **Outcome of MDR-TB patients, 1983-1993. Prolonged survival with appropriate therapy**. *Am J Respir Crit Care Med 1996*, 153(1):317–324.

[13] National Tuberculosis Control Program of Vietnam : *Annual review meeting report of the National TB Control Programme of Vietnam, 2013.* Hanoi: National Tuberculosis Control Programme of Vietnam; 2013. Ref Type: Report.

[14] Pandey S, Quelapio M, Hennig C, Khanh PH: *Green Light Committee Monitoring Mission Report for Vietnam.* The Philippines: World Health Organization -Western Pacific Regional Office; 2013: 1-73

[15] Ministry of Health of Vietnam: *Approving the development plan for tuberculosis and lung diseases control network, period from 2011-2020.* Hanoi: Ministry of Health; 2011: 2357/QĐ-BYT.

[16] Osman M, Simpson J, Caldwell J, Bosman M, Nicol MP: **GeneXpert MTB/RIF version g4 for identification of rifampin-resistant tuberculosis in a programmatic setting**. *J. Clin. Microbiol. 2014; 52(11): 635-637*

[17] Trajman A, Durovnil B, Saraceni V, Cordeiro M, Cobelens F, van den Hoff S: **High positive predictive value of Xpert in a low rifampicin resistance prevalence setting**. *J. Ersjournal. 2014; 44(6): 1711-1713*

Figure 1. Estimated number of MDR-TB patients among notified TB cases in Viet Nam, 2013

Figure 2. PMDT performance for case detection and enrollment of MDR-TB in Vietnam

Figure 3. The enrollment proportion into the second-line treatment program of MDR-TB cases among the estimated number of notifiable MDR-TB cases in 35 PMDT provinces in 2013. Data are presented per socio-economic region.

Table 1. Actual enrollment and estimated total number of MDR-TB cases nationwide and in 35 PMDT provinces (the numbers in brackets are for lower and upper CI)

Ser. No	Region	Number enrolled	All provinces		35 PMDT provinces		% estimated number of MDR-TB covered by PMDT provinces
			estimated number of MDR-TB cases	% enrolled	estimated number of MDR-TB cases	% enrolled	
1	Red River Delta	234	859 (565-1,139)	27.3 (20.5-41.4)	627 (414-830)	37.3 (28.2-56.5)	73.0%
2	North-East	0	339 (223-449)	0.0	189 (124 -252)	0.0	56.0%
3	North-West	0	53 (35 -71)	0.0	9 (6-12)	0.0	17.0%
4	Northern Central	54	466 (305-618)	11.6 (8.7-17.7)	338 (222-449)	16.0 (12.0-24.3)	72.7%
5	Southern Central Coast	84	405 (266 -536)	20.8 (15.7-31.5)	304 (200 -403)	27.6 (20.8-41.9)	75.2%
6	Central Highland	0	110 (72- 146)	0.0	21 (14 - 27)	0.0	18.8%
7	South -East	495	1,546 (1,035-2,036)	32.0 (24.3-47.8)	1,408 (944 -1,854)	35.2 (26.7-52.4)	91.1%
8	Mekong Delta	81	1,288 (854 -1,704)	6.3 (4.8-9.5)	1,086 (720 -1,436)	7.5 (5.6-11.3)	84.3%
	Total	948	5,065 (3,355 -6,700)	18.7 (14.1-28.3)	3,982 (2,643 -5,264)	23.8 (18.0-35.9)	78.6%

Table 2. Percentage of presumptive MDR-TB patients tested by regions

Ser. No	Region	Number of presumptive MDR-TB patients tested	All provinces		35 PMDT provinces	
			Estimated Number of presumptive MDR-TB patients	% tested	Estimated Number of presumptive MDR-TB patients	% tested
1	Red River Delta	801	2,519	31.8%	2,035	39.4%
2	North-East	263	1,154	22.8%	627	42.0%
3	North-West	43	214	20.1%	46*	93.9%
4	Northern Central	460	1,144	40.2%	886	51.9%
5	Southern Central Coast	668	1,117	59.8%	897	74.5%
6	Central Highland	0	290	0.0%	69	0.0%
7	South -East	2,917	6,991	41.7%	6,503	44.9%
8	Mekong Delta	516	4,736	10.9%	3,935	13.1%
	**Total**	**5,668**	**18,165**	**31.2%**	**14,998**	**37.8%**

** one PMDT province in North – West*

Table 3. Presumptive MDR-TB patients estimated and tested by risk category in the whole country and in 35 PMDT provinces of Vietnam 2013

**Presumptive category**		Estimate number for whole country*	Estimate number for PMDT areas only	Number of Xpert tests performed	% tested in PMDT areas
**Retreatment**	Failure**	593	519	711	ND
	Relapse	7,059	5,673	2,641	46.6%
	Defaulters	472	376	129	34.3%
	Other	739	662	210	31.7%
**Non-converters at 2 and 3 months**	New	2,713	2,214	290	13.1%
	Retreatment	586	482	413	85.7%
**MDR contacts**		402	402	249	62.0%
**TB/HIV**		3,828	3,224	340	10.5%
**>1 month using TB drugs**		1,773	1,446	685	47.4%
**Total MDR-TB suspects**		18,165	14,998	5,668	37.8%

** The denominator is the number of presumptive by categories that need to be tested.*

*** Failure is defined as sputum smear positive at 5 months or later during treatment, so one failure case could receive one or 2 tests (at 5 and/or 7 months). ND: not done as failure cases can be tested on multiple occasions.*

Table4. Obstacles to enrolling MDR-TB patients for treatment and solutions for an effective PMDT

Ser. No	Obstacle	Proposed solution
1	A set of key documents is lacking: updated guidelines, concise and clear SOPs, and standard training modules.	The NTP is strongly recommended to ensure that key documents are prepared and circulated to appropriate staff, with proper training. Letters with updates should be discouraged, unless there is an urgency.
2	Failures in identifying presumptive MDR-TB cases for screening.	The development of consistent training modules in accordance with national guidelines and SOPs.
3	Absence of a sound referral system for sending sputum samples to a laboratory.	A sound national referral system should be set up with a shipping agency who can do this safely.
4	Current Vietnam policy is to require patient to be hospitalized at the start of treatment. However, there is insufficient hospitalization capacity and patients may refuse to be referred to another treatment centre in another province either due to distance from hometown or additional costs without getting health insurance reimbursement.	The NTP policy needs consider the adoption for ambulatory treatment with community based care as also recommended by WHO [3]. Health insurance need to support MDR-TB patients in reimbursing costs in case there is a need for referral to another province for PMDT treatment.
5	Temporary MDR-TB drug stock-out due to procurement and distribution delay resulted in patients either not enrolled for treatment or a delay in treatment.	Improve drug procurement and distribution system.
6	TB units in many districts remain located in health centers that focus on prevention, and are separate from the general hospitals, which is discrepant from MoH policy.	Enforce policy to locate TB units in the district general hospitals. Training should be provided to appropriate staff.
7	Poor links between the NTP and public sector and no management system for MDR-TB patients in prison. There is no mechanism to refer MDR-TB presumptives or MDR-TB patients from the private sector to the NTP to be diagnosed or for treatment. The private sectors often do not notify MDR-TB cases to the NTP.	Establish the collaboration between the private sector and PMDT. Ensure private sector adheres to treatment guidelines for TB and MDR-TB. Provide diagnosis and treatment service for MDR-TB patients in prison.

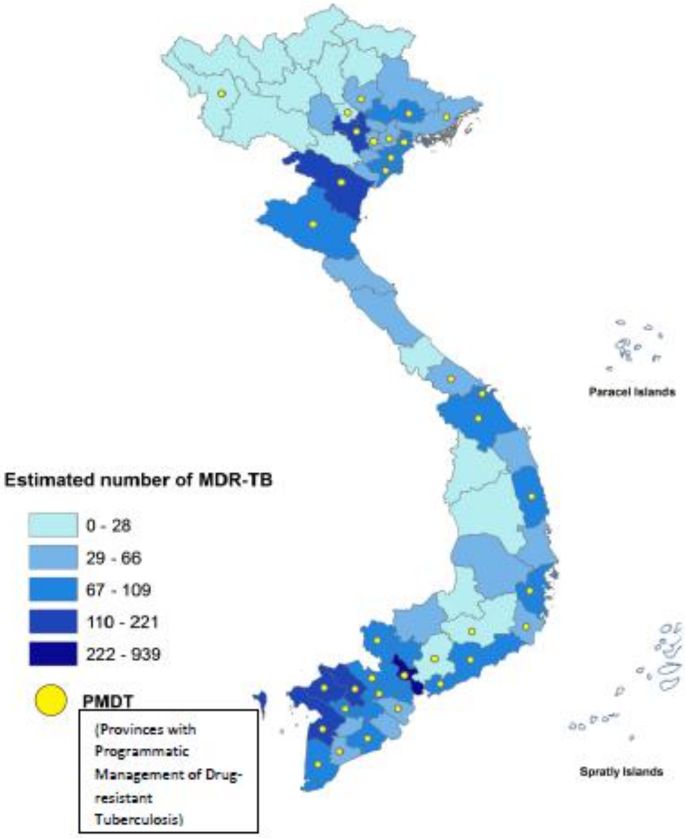

Paracel Islands
Spratly Islands
Estimated number of MDR-TB
0 - 28
29 - 66
67 - 109
110 - 221
222 - 939
PMDT
(Provinces with Programmatic Management of Drug-resistant Tuberculosis)

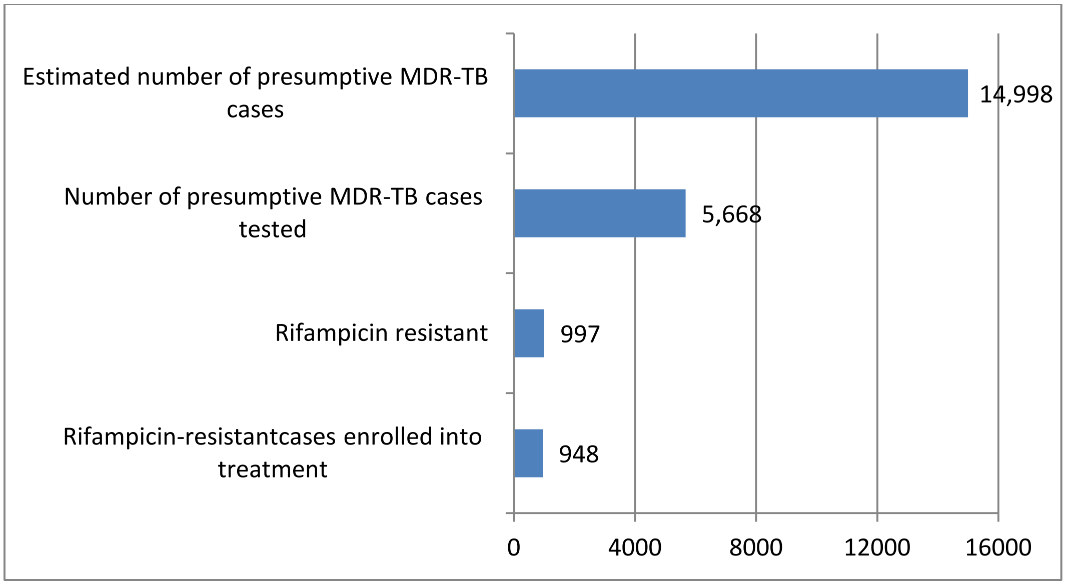
Estimated number of presumptive MDR-TB cases
Number of presumptive MDR-TB cases tested
Rifampicin resistant
Rifampicin-resistantcases enrolled into treatment
14,998
5,668
997
948
0
4000
8000
12000
16000

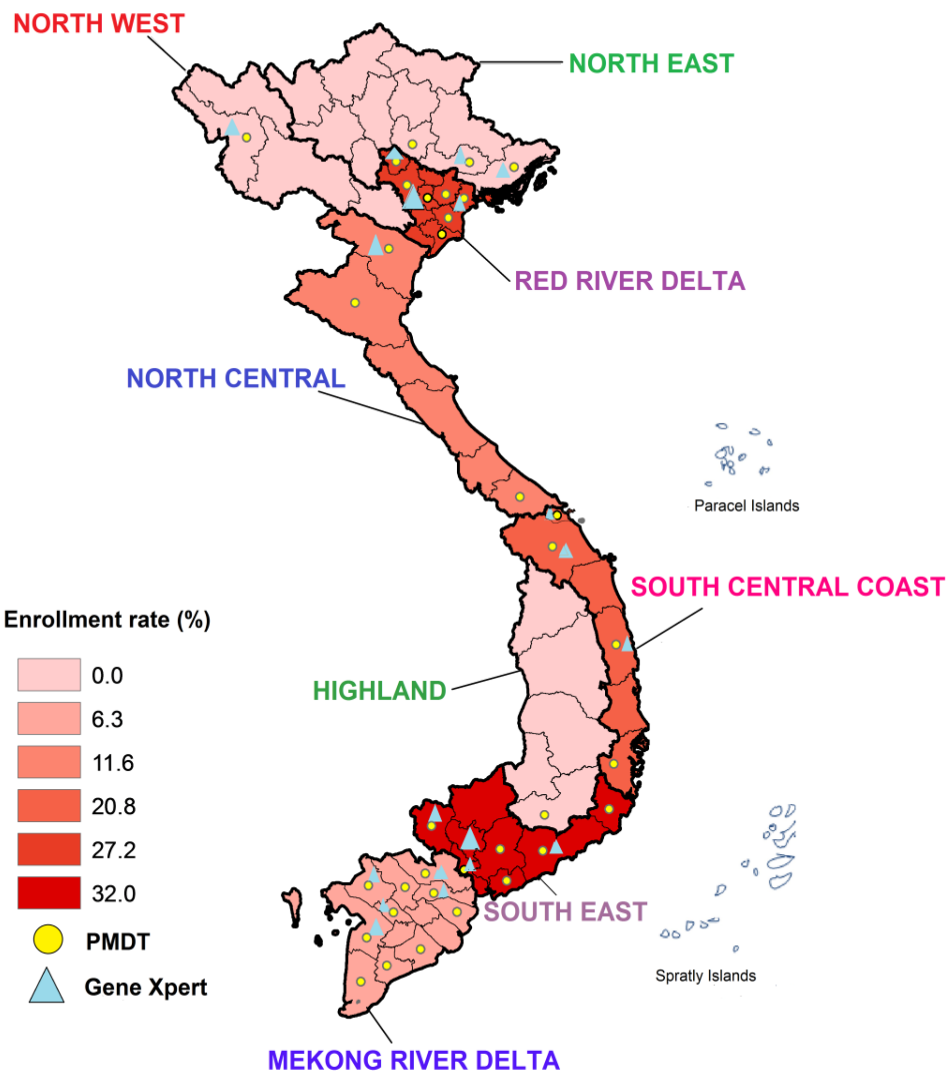
NORTH WEST
NORTH EAST
RED RIVER DELTA
NORTH CENTRAL
Paracel Islands
SOUTH CENTRAL COAST
Enrollment rate (%)
0.0
6.3
11.6
20.8
27.2
32.0
PMDT
Gene Xpert
HIGHLAND
SOUTH EAST
Spratly Islands
MEKONG RIVER DELTA
